# An MRS-YOLO Model for High-Precision Waste Detection and Classification

**DOI:** 10.3390/s24134339

**Published:** 2024-07-04

**Authors:** Yuanming Ren, Yizhe Li, Xinya Gao

**Affiliations:** College of Science, Qingdao University of Technology, 777 Jialingjiang East Road, Huangdao District, Qingdao 266520, China; sempusem@outlook.com (Y.R.); liyizheok@gmail.com (Y.L.)

**Keywords:** YOLO, dynamic convolution, waste detection, small target detection

## Abstract

With the advancement in living standards, there has been a significant surge in the quantity and diversity of household waste. To safeguard the environment and optimize resource utilization, there is an urgent demand for effective and cost-efficient intelligent waste classification methodologies. This study presents MRS-YOLO (Multi-Resolution Strategy-YOLO), a waste detection and classification model. The paper introduces the SlideLoss_IOU technique for detecting small objects, integrates RepViT of the Transformer mechanism, and devises a novel feature extraction strategy by amalgamating multi-dimensional and dynamic convolution mechanisms. These enhancements not only elevate the detection accuracy and speed but also bolster the robustness of the current YOLO model. Validation conducted on a dataset comprising 12,072 samples across 10 categories, including recyclable metal and paper, reveals a 3.6% enhancement in mAP50% accuracy compared to YOLOv8, coupled with a 15.09% reduction in volume. Furthermore, the model demonstrates improved accuracy in detecting small targets and exhibits comprehensive detection capabilities across diverse scenarios. For transparency and to facilitate further research, the source code and related datasets used in this study have been made publicly available at GitHub.

## 1. Introduction

As the global economy continues to advance and technology progresses, addressing environmental concerns becomes increasingly crucial. However, effectively categorizing social waste remains a significant challenge [1]. Waste separation persists as a prominent issue in society, characterized by labor-intensive processes [2], diverse waste types, and resource wastage. Manual sorting [3], still prevalent today, is hindered by inefficiency and high costs, posing obstacles to recycling efforts [4]. Therefore, there is an urgent need for intelligent, automated technologies to enhance sorting speed while maintaining precision, which is critical for optimizing recycling and environmental protection [5].

Existing waste detection and recycling systems primarily rely on target detection algorithms to identify garbage [6]. These algorithms are mainly categorized into two-stage and one-stage approaches [7]. Two-stage approaches, including R-CNN, Mask R-CNN, and Faster-RCNN [8], have demonstrated success in target detection but possess numerous shortcomings when applied to garbage detection and classification. The two-stage model requires preliminary segmentation of candidate regions for subsequent classification and localization, significantly prolonging the overall reasoning time due to its multi-stage process [9]. Additionally, given the diversity in garbage detection, numerous small target detection tasks abound, escalating computational overhead and imposing significant computational burdens [10].

Compared to traditional two-stage algorithms, one-stage approaches such as YOLO, SSD, and CornerNet [11], and their variants offer a significant advantage: the capability to simultaneously detect multiple targets within an image [12]. This feature is pivotal for classification systems tasked with rapidly processing vast amounts of garbage. Among them, SSD is more effective in detecting small targets through multi-scale detection mechanisms and prediction on feature maps of different scales, but it tends to produce a large number of redundant candidate frames [13]. CornerNet introduces a novel approach to localization via key points, enhancing its ability to detect various poses of similar targets with remarkable robustness [14]. However, its detection performance tends to diminish in dense detection scenarios typical of garbage detection. YOLO (You Only Look Once) has emerged as a popular target detection algorithm, efficiently detecting small targets with faster detection speeds compared to other methods [15]. However, its accuracy slightly trails behind that of two-stage detection models [16]. Additionally, YOLO demonstrates inferior robustness and migration generalization capabilities compared to CornerNet [17].

Currently, the YOLO series enjoys an adoption rate exceeding 80% in garbage detection tasks, primarily focusing on surface-level garbage detection with limited research on municipal garbage recycling [18]. For instance, Yang et al. proposed a YOLOv5-based model for detecting floating garbage in rivers, achieving an 88.5% detection accuracy [19]. Similarly, Chen and Zhu introduced a lightweight YOLOv5 detection model, enhancing detection speed by 4.3% and achieving an mAP value of 84.9% [20] García et al. (2022) proposed an improved YOLOv4 model specifically tailored for detecting small-sized waste items in urban environments, achieving a precision of 90.2% with an mAP of 86.7% [21]. Liu et al. (2021) introduced an advanced version of YOLOv3, named YOLOv3-SPP, to improve the detection of small and densely packed waste objects, reporting an mAP of 87.3% and a detection speed of 45 FPS [22]. Zhang et al. (2021) developed a YOLOv4-based model optimized for detecting mixed waste in complex backgrounds, resulting in a detection accuracy of 89.4% and an mAP of 85.2% [23]. Wang et al. (2022) enhanced the robustness of YOLOv5 for real-time waste detection in various lighting conditions, achieving an mAP of 88.6% and a precision of 91.0% [24]. Li et al. (2022) proposed a hybrid YOLOv5 and Transformer-based model to address the challenge of detecting small and overlapping waste items in recycling facilities, demonstrating an improvement with an mAP of 89.8% and a recall rate of 92.1% [25]. Although research on garbage detection models mainly focuses on improving detection accuracy and speed, there is still relatively little research on the small target detection capabilities and robustness of these models.

To address the challenge of classifying urban garbage effectively, this paper proposes an urban garbage detection model based on the YOLO framework. Compared to the original garbage detection model, this model enhances small target detection and overall model generalization ability. Firstly, the paper introduces improvements to the SlideLoss_IOU method, enhancing the recognition of small targets. Secondly, to tackle the YOLO model’s poor robustness, the paper enhances the C2f feature extraction method in YOLOv8 by employing a multidimensional convolution approach instead of the original convolution method [26]. Additionally, a dynamic convolution method is adopted to flexibly extract features from different feature maps obtained through convolution [27]. Furthermore, to bolster the model’s detection capability, the paper incorporates a Transformer mechanism to optimize feature utilization while reducing computational costs [28]. This model outperforms traditional detection models in terms of robustness, inference speed, and detection accuracy.

## 2. Modeling

This paper introduces MRS-YOLO, a novel garbage detection model designed to improve small target detection, feature extraction, and feature utilization. The contributions of this work are presented across four key components: the model framework, small target detection methodology, an enhanced feature extraction approach, and the integration of a Transformer mechanism.

### 2.1. Model Framework

The primary distinctions between YOLOv8 and YOLOv5 lie in their architectural enhancements. YOLOv8 introduces the C2f module, inspired by the CSP concept [29], as a replacement for the C3 module. Additionally, YOLOv8 employs a dynamic TaskAlignedAssigner [30] strategy for matching tasks.

The C2f module in YOLOv8 incorporates the ELAN concept from YOLOv7, merging C3 and ELAN to create a unified module. This integration enriches YOLOv8 with more comprehensive gradient flow information while maintaining its lightweight design. Additionally, the backbone still incorporates the widely used SPPF module, where three consecutive Maxpools with a size of 5 × 5 are applied at the end. These layers are connected in series to ensure accurate object detection across various scales while preserving the model’s lightweight nature.

At the neck, YOLOv8 maintains the utilization of the PAN-FPN feature fusion method [31], which enhances the integration and exploitation of information across feature layers of different scales. YOLOv8’s neck module consists of two upsampling layers, multiple C2f modules, and a final decoupled head structure. Inspired by YOLOx, YOLOv8 introduces the concept of decoupling the head for the final part of the neck, effectively combining confidence and regression frames to achieve heightened accuracy.

**Figure 1:**
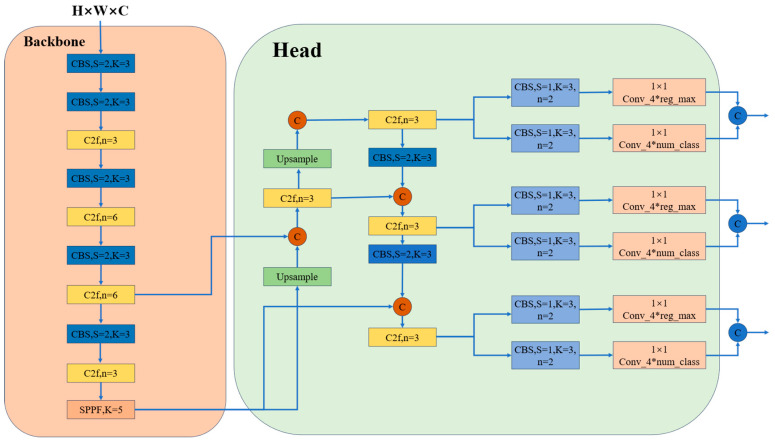
YOLOv8 model structure.

YOLOv8 offers support for all YOLO versions and allows seamless switching between them. Moreover, it is compatible with various hardware platforms, including CPU and GPU, rendering it highly versatile. The network architecture of YOLOv8 is depicted in Figure 1.

### 2.2. SlideLoss_IOU Methods

In real-world garbage detection tasks, detecting small garbage samples with high accuracy poses [32] a challenge, especially considering the prevalence of small domestic garbage items [33]. The SlideLoss method addresses the issue of highly skewed distribution of sample types within the dataset. For instance, certain garbage types like plastic bottles or paper may occur frequently, while others like hazardous waste are comparatively rare. To prioritize the detection of these less common samples, SlideLoss increases penalty weights for them. The underlying principle of SlideLoss is illustrated in Equations (1) and (2).

(1) SlideLossy,y^,u=ωu⋅Ly,y^

(2) $$w\left(u\right)=\left\{\begin{array}{c} 1\;x\leq \mu -0.1\\ e^{1-\mu }\;\mu <x<\mu -0.1\;\\ e^{1-x}\;x\geq \mu \; \end{array}\right.$$

**Figure 2:**
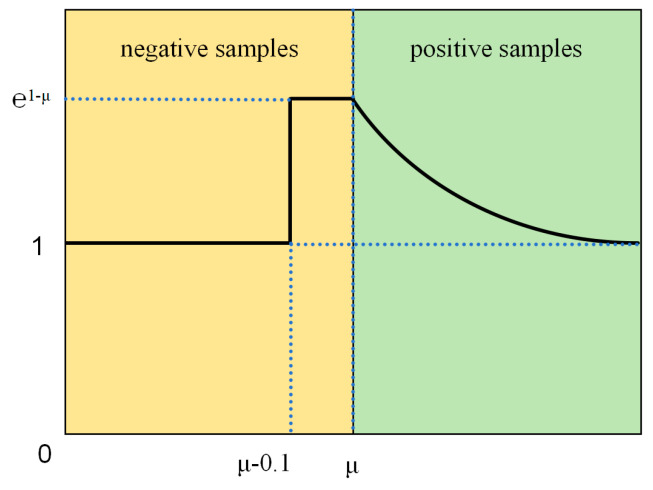
SlideLoss method weight variation.

In Equation (1), L(y,y^) represents the loss function, where y denotes the true value and y^ signifies the model’s predicted value; $\omega \left(u\right)$ denotes a weight function, with u representing the average value of Intersection over Union (IoU). SlideLoss dynamically adjusts the loss of individual samples based on their IoU values, thereby improving the model’s capacity to identify and classify challenging samples. This intuitive adjustment mechanism is depicted in Figure 2.

In this paper, a novel small-objective strategy (SlideLoss_IOU) is introduced, building upon the concept of escalating penalty weights. Unlike traditional SlideLoss, which adjusts sample weights using a fixed threshold, SlideLoss_IOU refines the loss function by dynamically adapting the loss weights based on Intersection over Union (IoU). The underlying principle is elucidated in Equations (3)–(5) as follows.

(3) IOUmean=d∗IOUmean+1−d∗autoIOU^

(4) $$d=decay\times \left(1-e^{-\frac{x}{\tau }}\right)$$

(5) μ=IOUmean

**Figure 3:**
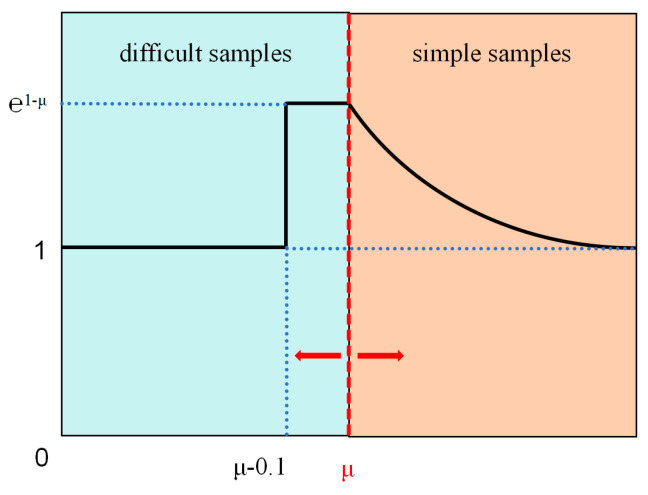
SlideLoss_IOU method weight variation.

Here, IOUmean represents the calculated mean value of IOUs. The value autoIOU^ is solely utilized for computation and does not partake in gradient descent or echelon descent. d denotes the decay factor, while decay serves as an artificial parameter controlling the decay rate. τ represents a time constant regulating the decay rate, with x denoting the number of training iterations. Following computation, the computed IOUmean is assigned to autoIOU as the new threshold, as depicted in Figure 3 for clarity.

Unlike the traditional method, which addresses the imbalance in sample distribution by fixing the threshold, the approach presented in this paper dynamically adjusts the threshold value, thereby allocating more focus to small target detection. Additionally, this method exhibits enhanced flexibility in scenarios involving occlusion, thereby augmenting the model’s detection capability.

### 2.3. Channel Pooling with Dynamic Convolution Method

To address the issue of poor robustness in YOLO for garbage detection, this paper introduces a novel feature extraction module called Channel Pooling with Dynamic Convolution (CP), based on the C2f module of YOLOv8 [34]. The CP module is developed by enhancing the functionality of C2f. While preserving the core functionalities of C2f, the CP module employs a multi-dimensional pooling convolution approach to replace the original single convolution module. Additionally, a dynamic convolution mechanism is proposed to apply distinct convolution strategies to different feature maps obtained from convolution. These enhancements ensure that the model maintains robust target detection capabilities across various categories of garbage detection and exhibits greater flexibility in handling different feature maps.

**Figure 4:**
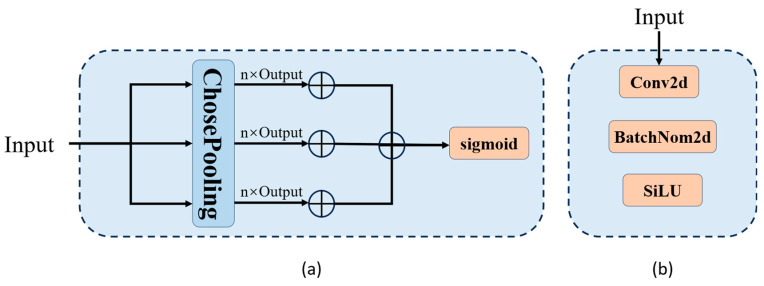
Convolutional comparison: (**a**) Structure proposed in this paper. (**b**) C2f feature module.

In the convolutional component, this paper introduces a novel approach, PoolAttention, for feature extraction, contrasting with the Conv module utilized in C2f. The structure of PoolAttention is compared with the original convolutional structure of C2f, as depicted in Figure 4. The essence of this module lies in dimensionally transforming the input feature map to acquire three distinct perspectives. Subsequently, pooling operations are performed on each perspective, and the results are linearly summed before being passed through an activation function to produce the output. This process aims to prevent overfitting, enhance model robustness, and ensure flexibility. The underlying principle is elucidated in Equations (6)–(8).

(6) $$PoolingOut=\sum_{Pooling}^{\left[Avg,max,std\right]}Pooling\left(Input\right)$$

(7) $$Out=\sum_{Pooling}^{\left[Avg,max,std\right]}Pooling\left(Input\right)\times \omega +\frac{1}{n}\cdot \left(PoolingOut\right)\omega \in \left[0,1\right]$$

(8) $$sigmoid\left(x\right)=\frac{1}{1+e^{-x}}$$

In Equation (7), ω represents the weights associated with different pooling operations. These weights play a crucial role in significantly reducing the computational cost of subsequent operations. By incorporating various pooling operations and introducing an adaptive attention mechanism, the PoolAttention module dynamically adjusts and optimizes different dimensions of the feature map in a detailed manner. This enables the extraction of richer and more effective feature representations, enhancing the overall performance of the model.

The C2f method initiates with a slicing operation on the feature map before constructing the residual block. This slicing divides the feature map into segments, each representing a subset of features across different channels. This technique enhances the learning of inter-channel feature relationships, thereby boosting the network’s flexibility and expressiveness. However, the fixed number of cuttings in C2f limits its adaptability to varied data types. To address this, the article proposes an improvement by calculating the standard deviation of each feature map obtained during the convolution process to ascertain its feature distribution. Based on this, the number of segments is dynamically adjusted using a standard deviation threshold derived from training. The methodology for calculating standard deviation is detailed in Equations (9)–(11).

(9) $$\mu_{i}=\frac{1}{n}\sum_{j=1}^{n}x_{ij}$$

(10) $$\delta_{i}^{2}=\frac{1}{n}\sum_{j=1}^{n}(x_{ij}-\mu_{i}{)}^{2}$$

(11) $$\delta i=\sqrt{\delta_{i}^{2}}$$

**Figure 5:**
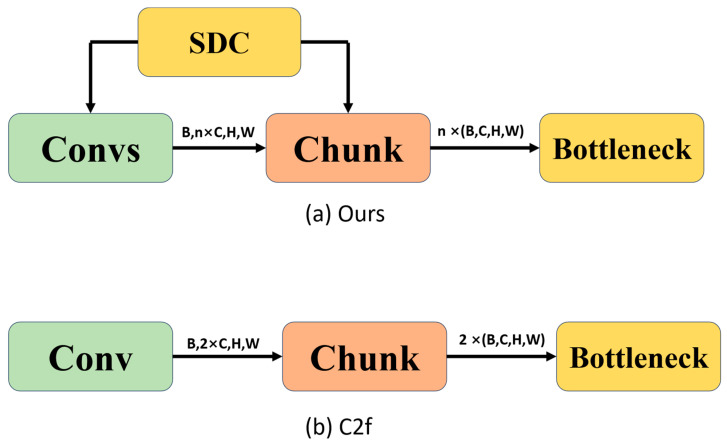
Comparison of feature extraction step slicing methods.

In Equation (9), $\mu_{i}$ represents the mean value of each channel i, while in Equation (11), $\sigma_{i}$ denotes the variance of each channel. The structure of the proposed method with C2f is illustrated in Figure 5.

By defining an appropriate threshold, the features within the feature map are categorized into three groups: sparse features, typical features, and abundant features. Consequently, the slicing strategy is adjusted accordingly to match the corresponding number of Bottleneck iterations. This adaptive approach substantially reinforces the model’s robustness, enabling it to adeptly handle diverse situations. Moreover, it dynamically adjusts the network structure based on the input data’s characteristics, thereby better aligning with various scenarios and task demands.

Precisely, the standard deviation of the feature map serves as a criterion for identifying the presence of abundant features. If the feature map contains rich features, it undergoes segmentation into simpler feature map parts, followed by multiple Bottleneck iterations to facilitate enhanced feature extraction. Conversely, if the features are sparse, the number of segmentations is decreased to preserve more global features, thereby mitigating computational costs to some extent. This adaptive strategy optimizes feature extraction while balancing computational efficiency.

**Figure 6:**
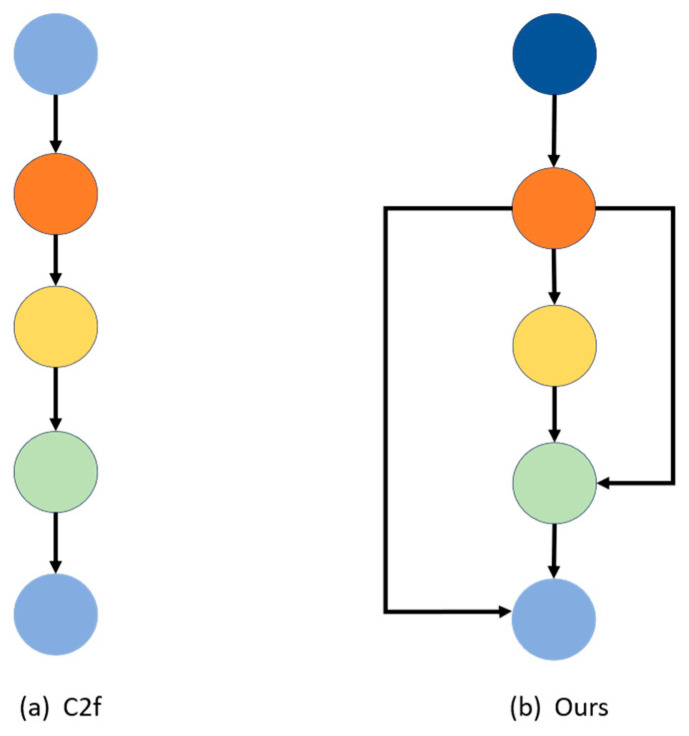
Module architecture comparison.

The C2f (Channel to Feature) technology of YOLOv8 draws inspiration from the ELAN mechanism and seamlessly integrates it with the C3 module. This fusion significantly elevates the model’s capacity to capture subtle features through channel expansion and feature re-decomposition, as illustrated in Figure 6a. Moreover, this method implements a dynamic feature map-cutting strategy (SDC) based on the standard deviation of input features. It calculates the standard deviation of each input feature map and dynamically adjusts the number of cuts according to a specified threshold. This allows the model to dynamically adapt its processing flow to changes in input data, enhancing flexibility across different scenarios, as depicted in Figure 6b.

This strategy not only streamlines the feature extraction process but also bolsters the model’s robustness and generalization ability when faced with various data types. Through these innovations, this research aims to push the boundaries of the YOLO model in terms of feature extraction efficiency and robustness in garbage detection, providing novel solutions for garbage detection tasks across diverse data categories.

### 2.4. RepViT Network Architecture Optimization Method

To tackle complex litter detection tasks more effectively and leverage global features efficiently, this paper references an enhanced Transformer visual model called RepViT [35]. Traditional Transformer models experience a quadratic increase in computational and memory requirements with the sequence length, making them less suitable for tasks involving complex scenes and diverse targets. The RepViT module addresses this challenge by incorporating a reparameterization technique, optimizing the Transformer’s structure to reduce computational costs and processing time. This adaptation renders the Transformer model more conducive to object detection tasks.

**Figure 7:**
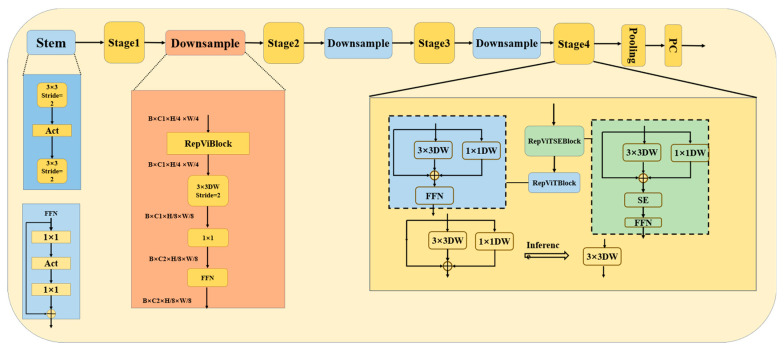
RepViT structure.

In the RepViT architecture, depicted in Figure 7, the input image undergoes feature extraction via a 3 × 3 convolutional kernel in the initial layer (Stem), halving its spatial dimensions. Subsequently, in each subsequent stage (stage 1 to stage 4), one or more RepViT modules are employed for computation. Between adjacent stages, a downsampling step further reduces the spatial dimensionality of the image while increasing its depth, enabling a focus on higher-level features and mitigating computational overhead.

**Figure 8:**
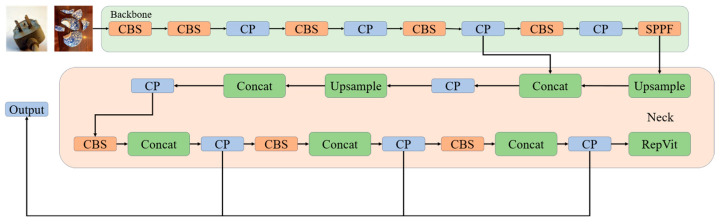
MRS−YOLO overall structure.

By integrating the RepViT architecture into our model, we harness the Transformer’s self-attention mechanism to capture long-range dependencies within the image. Additionally, the reparameterization technique mitigates the risk of overfitting on smaller datasets, enhancing the model’s robustness and accuracy. This combined approach provides a more effective solution to the intricate task of litter detection. Finally, the overall model structure is shown in Figure 8.

### 2.5. Overview of the MRS-YOLO Model

The MRS-YOLO model introduced in this paper is an advanced waste detection and classification system that significantly enhances model performance and flexibility through a series of technological innovations. The core architecture of the MRS-YOLO model includes an improved feature extraction module, optimized loss function, and efficient feature utilization mechanisms, designed to address various challenges encountered in practical applications.

The backbone and neck parts of the model employ the newly developed Channel Pooling with Dynamic Convolution (CP) module, which innovatively combines multidimensional pooling and dynamic convolution technologies for efficient feature extraction and dynamic parameter adjustment. Multidimensional pooling utilizes different types of pooling operations (such as max pooling, average pooling, and standard deviation pooling) to provide richer feature descriptions than single pooling, capturing more detailed image layers, effectively enhancing the model’s adaptability to input variations. Simultaneously, the dynamic convolution technology dynamically adjusts its parameters based on different input features. This flexibility allows the model to better adapt to data diversity, optimizing convolution operations and reducing unnecessary computations. The combination of these technologies not only increases the efficiency of feature map processing but also significantly enhances the model’s detection capabilities across different types of waste, reducing computational costs while maintaining high performance.

In terms of the loss function, this paper innovatively proposes the SlideLoss_IOU method, which dynamically adjusts the loss weights based on the Intersection over Union (IoU), particularly enhancing the model’s ability to detect small and rare waste samples. This enables MRS-YOLO to more accurately identify and classify small targets that are often overlooked by traditional models.

Furthermore, to enhance feature utilization efficiency and reduce computational costs, the model integrates the RepViT module based on the Transformer mechanism in the final part of the neck. This module not only optimizes the flow of information and integration of features but also effectively manages long-distance dependencies through its self-attention mechanism, significantly improving detection accuracy and speed.

Overall, the MRS-YOLO model provides an efficient, precise, and robust solution for waste classification and detection tasks through these structural innovations. The overall structure of the model is illustrated in Figure 8. The model’s design takes into account the needs of practical applications, particularly excelling in handling diverse and complex types of waste. By making the source code and related datasets publicly available, this research not only advances waste detection technology but also contributes to environmental conservation efforts.

## 3. Experimental Design

### 3.1. Data Sets and Preprocessing

The dataset used in this study contains 12,072 high-definition images (before data augmentation) covering 10 different trash categories. The data collection process includes several steps to ensure the diversity and authenticity of the dataset. First, a total of 14,964 trash photos were taken using a smartphone under different lighting conditions and backgrounds (all uploaded to GitHub), and then 9285 photos were randomly selected from them.

The specific lighting conditions include natural light taken outdoors during the day, shadows taken in partially shaded areas to simulate different outdoor scenes, and night lighting taken under artificial lighting conditions at night to test the performance of the model in low light.

To further ensure the generalization ability of the model, different background types are included. The backgrounds vary: indoor images taken in buildings with various indoor settings, outdoor images taken in open spaces such as parks, streets, and campuses, cluttered backgrounds to test the model’s ability to distinguish between trash and complex backgrounds, and simple backgrounds taken on flat surfaces to focus on trash.

Each photo goes through a meticulous manual labeling process to determine the trash category and create bounding boxes for each object. This process is performed using the standard image labeling tool LabelImg. The specific steps involved include carefully inspecting each image to identify and classify trash, creating bounding boxes around each piece of trash using LabelImg, assigning trash items within each bounding box to one of 10 predefined categories, and performing secondary checks to ensure the accuracy and consistency of labeling.

To prepare the images for the model, several preprocessing steps were applied. The images were resized to fit the model input requirements to ensure consistency. Pixel values were normalized to increase the convergence rate of the model training process. Various data augmentation techniques were employed, including rotating at random angles, cropping parts of the image to simulate different perspectives, color inversion to increase diversity, and splicing parts of different images to create new training samples. These steps were implemented to enhance the generalization ability of the model and ensure robust performance in diverse and realistic scenarios. The specific model training steps will be specified in the Appendix A.

### 3.2. Experimental Design

**Figure 9:**
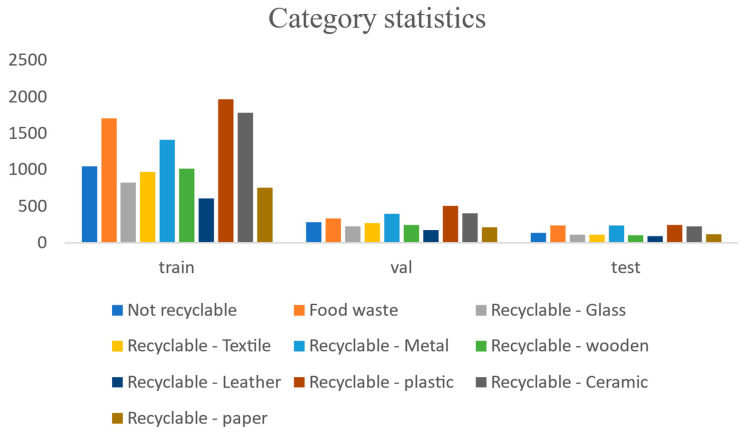
Distribution of data categories in validation, training, and testing.

**Figure 10:**
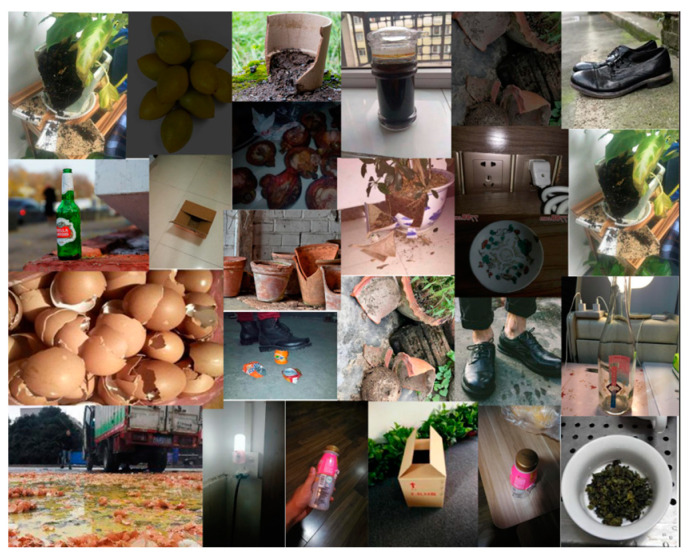
Sample of selected datasets.

For the partitioning of training, testing, and validation datasets, this study randomly divides the overall dataset into training (70%), testing (20%), and validation (10%) sets, ensuring that the distribution of categories across the three subsets closely mirrors that of the entire dataset. The category distribution of the dataset is depicted in Figure 9, while Figure 10 illustrates examples from the dataset.

In terms of evaluation metrics, this paper employs accuracy, recall, F1 score, and mean average precision (mAP) as the primary assessment criteria to comprehensively evaluate the model’s performance. Equations (12) and (13) illustrate these metrics.

(12) $$P=\frac{TP}{\left(TP+FP\right)}$$

(13) $$R=\frac{TP}{\left(TP+FN\right)}$$

TP represents the count of correctly predicted bounding boxes, while FP indicates the number of incorrectly classified positive samples, and FN denotes the quantity of undetected targets. The average precision (AP) refers to the mean precision of the model, with AP being the average value of AP across all categories (K). The calculation of AP and mAP involves the formulae depicted in Equations (14) and (15).

(14) $$AP=\int_{0}^{1}P\left(x\right)dx$$

(15) $$mAP=\frac{1}{n}\sum_{i=1}^{n}APi$$

The training hardware environment consists of a 24 GB RAM NVIDIA GTX4090 GPU, with the software environment comprising Ubuntu20, torch2.12, cuda11.8, and Anaconda. The training process utilizes the Adam optimizer with an initial learning rate set to 1 × 10^−4^, incorporating learning rate decay and early stopping strategies to mitigate overfitting. The batch size and training duration were fine-tuned based on preliminary experimental findings. The algorithm underwent iterative training and testing on the dataset, optimizing each stage and comparing results with mainstream YOLO series models. Through numerous experiments, it was determined that the algorithm typically converges after 200 iterations. Subsequently, adjustments were made to hardware settings and several experimental iterations were conducted, leading to the following parameters: batch size of 16 and training epochs set to 300.

## 4. Experimental Verification

The model’s performance is assessed within the experimental framework outlined above to validate the accuracy and robustness improvement capabilities of the proposed CP, SlideLoss_IOU, alongside the introduced RepViT network architecture for litter detection. Concurrently, a comparative analysis is conducted with existing YOLO litter detection models to showcase the strengths and contributions of this research endeavor.

### 4.1. Comparison Experiment

To validate the effectiveness of the enhancements introduced in the MRS-YOLO model, a comparative test was conducted with other widely recognized litter detection models under identical control parameters. The target detection algorithms selected for comparison included YOLOv3, YOLOv5n, YOLOv5s, YOLOv6n, and YOLOv8n. All models were evaluated using the same equipment, dataset, and training parameters. The comparison results are summarized in Table 1 below.

The results reveal that our proposed model, based on the Darknet-53 with Channel Pooling (CP) backbone, outperforms the others in terms of mAP at both IOU thresholds, achieving a higher accuracy of 74.5% at IOU 0.5 and 65.5% at IOU 0.5:0.95. This superior performance can be attributed to several key innovations.

The Channel Pooling with Dynamic Convolution (CP) modules significantly enhance feature extraction by performing multidimensional pooling and dynamic convolution operations. This approach allows the model to adaptively extract features from various feature maps, capturing more detailed and diverse features. As a result, our model achieves higher mAP scores compared to others, particularly in detecting small and complex objects. The dynamic convolution mechanism ensures that both prominent and subtle features are effectively captured, leading to superior detection accuracy.

Additionally, the SlideLoss_IOU technique dynamically adjusts loss weights based on Intersection over Union (IoU) values, prioritizing difficult-to-detect small objects. This adjustment ensures that small objects receive more attention during training, improving the model’s ability to accurately detect these targets. The enhanced performance in mAP0.5:0.95 indicates the model’s improved accuracy in detecting small objects, which is a common challenge in waste detection tasks.

Furthermore, the integration of the RepViT module leverages the Transformer mechanism to capture long-range dependencies within the image, providing a comprehensive understanding of spatial relationships. This capability is particularly beneficial in cluttered and complex scenes. The reparameterization technique reduces computational costs and processing time, making the model more efficient. As a result, our model achieves a competitive speed of 2.7 ms per frame, which is faster than other models like YOLO v5n and YOLOv5s that have higher processing times.

In conclusion, the MRS-YOLO model’s innovative design and excellent performance provide an effective solution for litter detection, setting a strong foundation for future research and applications in the field.

### 4.2. Ablation Experiment

The innovations of this paper encompass three main contributions: the proposal of a novel feature extraction method called MRS based on C2f, the introduction of a small target detection technique named SlideLoss_IOU, and the optimization of network architecture through the integration of RepViT. To validate the effectiveness of each component, ablation experiments were conducted, comparing the performance with that of the original model. The experimental findings are presented in Table 2.

The ablation experiments conducted to evaluate the effectiveness of the proposed innovations-CP, SlideLoss_IOU, and RepViT-reveal profound findings. When a component is added individually to the model, notable improvements in junk detection performance are observed. Specifically, integrating CP leads to a modest increase in mAP at both IOU 0.5 and 0.5:0.95, demonstrating its contribution to enhancing feature extraction efficiency. Adding SlideLoss_IOU results in a further enhancement of mAP values, underscoring its effectiveness in object detection. Moreover, the integration of RepViT not only improves the accuracy and speed of the model but also demonstrates the substantial impact of network architecture optimization. Most notably, when all three components are combined, the model achieves its highest mAP scores, highlighting the synergistic effect of these innovations. This combination significantly advances the state-of-the-art in waste detection technology, providing a robust, efficient, and highly adaptable solution for real-world applications.

The ablation experiments underscore the importance of each component in improving litter detection performance, validating the effectiveness of the proposed approach. This comprehensive evaluation reaffirms the potential of the proposed model in addressing the challenges of litter detection tasks and advancing the state-of-the-art in this domain.

**Figure 11:**
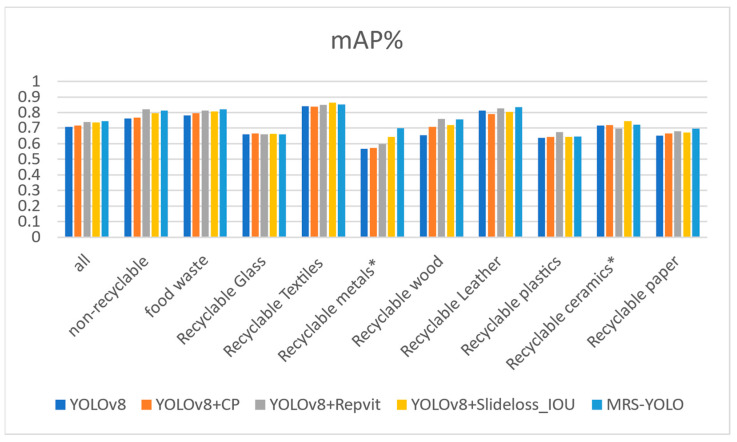
Accuracy in all categories. * Represents small target categories.

Figure 11 illustrates the category-wise accuracy improvements achieved by incorporating various modules into the ablation experiment. YOLOv8+CP denotes the results post the inclusion of the CP module to the YOLOv8 model, YOLOv8+RepViT refers to the outcomes following the addition of the RepViT module, YOLOv8+Slideloss_IOU represents the performance after integrating the SlideLoss_IOU technology, and MRS-YOLO represents the outcomes from our integrated model.

Across all categories, the MRS-YOLO model consistently outperforms other models, boasting an overall accuracy of 0.745, a notable enhancement of 0.036 over the baseline YOLOv8 model. Particularly noteworthy are the high accuracies achieved by the MRS-YOLO model in non-recyclable garbage (0.814), food waste (0.821), and recyclable glass (0.659) categories. Notably, in the category of recyclable textiles, the MRS-YOLO model demonstrates exceptional accuracy, reaching 0.852, showcasing significant improvements over other models.

Furthermore, our model exhibits remarkable accuracy in detecting small targets, such as recyclable metals and recyclable ceramics, thereby affirming its efficacy in small target detection. These results collectively underscore the superior performance and effectiveness of the MRS-YOLO model across various categories, thereby reinforcing its suitability for garbage detection tasks.

The ablation experiments conclusively demonstrate that each component of the MRS-YOLO model significantly enhances its performance. The CP module improves feature extraction efficiency, SlideLoss_IOU markedly enhances small target detection, and RepViT optimizes network robustness and processing speed. The combined effect of these components results in the highest accuracy and efficiency, validating the proposed approach. This comprehensive evaluation not only highlights the individual contributions of each component but also emphasizes their synergistic effect, thereby reinforcing the robustness and applicability of the MRS-YOLO model for real-world waste detection and classification tasks. These findings underscore the potential of the MRS-YOLO model to advance the state-of-the-art in waste management systems, providing a robust and efficient solution for practical applications.

### 4.3. Feature Extraction Comparison Experiments

The algorithm presented in this study aims to enhance the performance of the C2f module in YOLOv8, thereby improving detection accuracy and robustness while maintaining computational efficiency. To achieve this objective, control variables are employed to ensure consistency across experimental conditions. Within the framework of YOLOv5, experiments are conducted using three different feature extraction modules: C3, C2f, and CP. The experimental results, summarized in Table 3 provide insights into the comparative performance of these modules.

The results indicate that the C2f module significantly outperforms the C3 module in terms of mAP0.5 and mAP0.5:0.95, showcasing its effectiveness in enhancing detection accuracy, with a notable improvement in mAP0.5 by approximately 6.79 percentage points. Furthermore, the CP module demonstrates superior performance compared to both C3 and C2f in terms of mAP0.5, highlighting its potential to further improve detection accuracy. However, it is observed that the CP module experiences a slight decrease in mAP0.5:0.95 compared to the C2f module. This suggests that while CP enhances overall detection performance, it may exhibit some limitations in accurately detecting more challenging targets. Overall, these findings underscore the importance of feature extraction module selection in optimizing detection accuracy and efficiency within the YOLOv5 framework.

**Figure 12:**
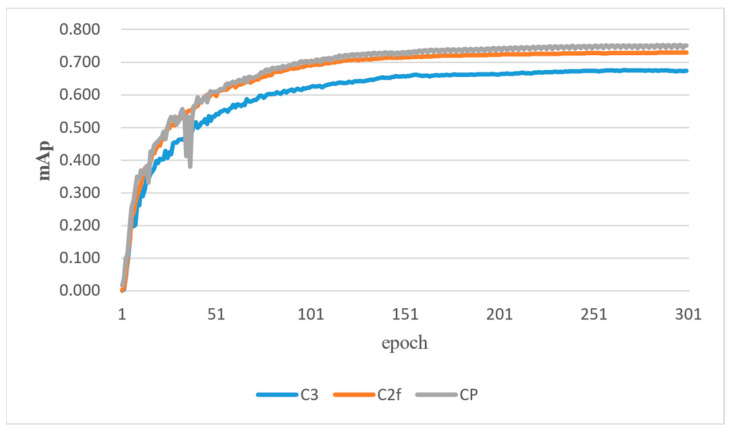
Comparison of experimental mAP for different feature extraction modules.

Based on the comparison of mean average precision (*mAP*) across different feature extraction modules (C2f, C3, and CP) throughout the training cycle as shown in Figure 12, the CP module consistently outperforms the others. After the training process, it achieves the highest final *mAP* value, nearly reaching 0.75. In contrast, the C2f module exhibits the second-highest *mAP* value, stabilizing around 0.69. The C3 module, on the other hand, demonstrates the lowest *mAP* value, approaching approximately 0.62 toward the end of training.

Indeed, the analysis highlights the superior performance of the CP feature extraction module in both feature extraction and model training. It demonstrates a remarkable capability to enhance the average accuracy of the model, outperforming the C3 and C2f modules by a significant margin.

### 4.4. Model Robustness Test

The standard deviation values calculated for the detection results of the YOLOv3, YOLOv5, YOLOv8, and our proposed model provide insights into the stability levels of their detection performance across different categories. A lower standard deviation indicates a more consistent and stable performance, while a higher standard deviation suggests greater variability in detection results.

**Figure 13:**
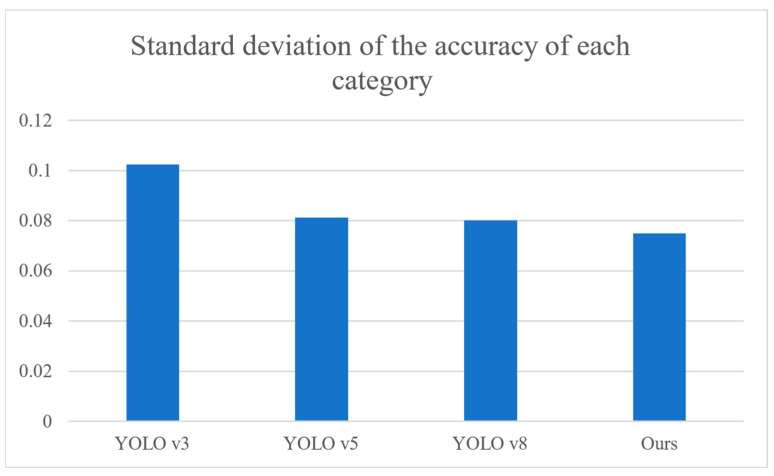
Standard deviations by category.

In the comparison result shown in Figure 13, our proposed model exhibits the lowest standard deviation of 0.074971, indicating a higher level of stability and consistency in detection across various categories compared to YOLOv3, YOLOv5, and YOLOv8. This implies that our model demonstrates a more reliable and robust performance in accurately detecting objects across different categories, which is crucial for applications requiring consistent detection performance in diverse scenarios. Additionally, the decreasing trend of standard deviation from YOLOv3 to YOLOv8 and further to our model suggests progressive improvements in stability and reliability as advancements are made in model architectures and techniques. Overall, the low standard deviation observed in our proposed model underscores its effectiveness in achieving stable and consistent detection results across different object categories, thereby enhancing its practical utility and reliability in real-world applications.

### 4.5. Multi-Environment Garbage Detection Test

To ensure the comprehensiveness of the evaluation and verify the stability of the model, the multi-environment garbage detection test was designed to cover a range of typical scenarios where the model may be deployed (including ordinary urban areas, suburban environments with different distribution of garbage spaces, and daily home environments) and different light source scenarios. To ensure the fairness of the experiment, all image samples used for prediction are from the open source datasets of the Visual China website (Visual China-the world’s leading visual material digital copyright library and trading platform (vcg.com)) and tacodataset.org. This approach helps to standardize the test conditions and ensure that any changes in model performance are attributed to the model itself, rather than external factors related to image quality or composition.

**Figure 14:**
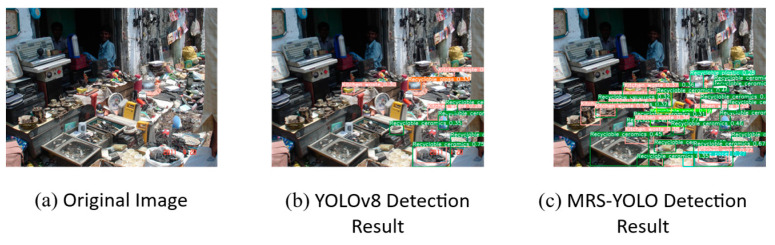
Comparison of detection effects of images with different scales (1).

**Figure 15:**
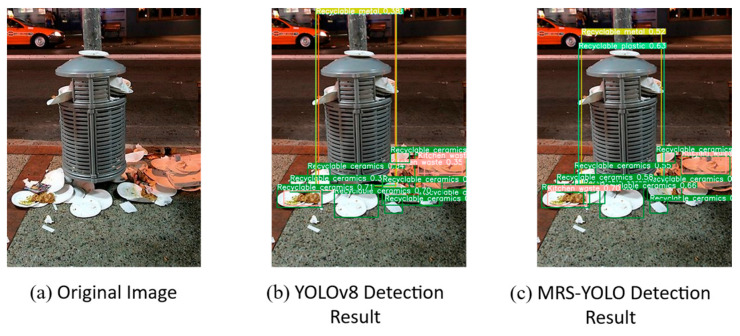
Comparison of detection effects of images with different scales (2).

This study evaluates the performance of two machine learning models, MRS-YOLO and YOLOv8, in the context of garbage detection across various environmental settings. Observations from Figure 14 and Figure 15 reveal that the MRS-YOLO model excels in detecting objects within complex backgrounds, such as market stalls and urban street scenes, demonstrating heightened accuracy and discrimination, particularly for small objects. The model’s sensitivity to detail becomes particularly evident when dealing with images of varying scales, an attribute critical for practical application.

**Figure 16:**
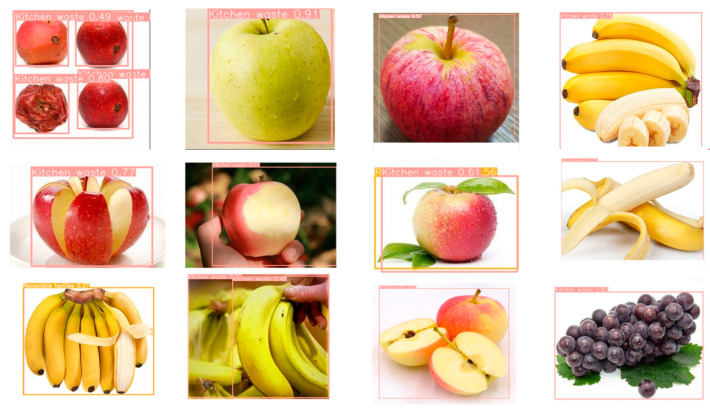
Test results of different types of food waste.

Additionally, it is important to emphasize that our research aims to identify the material and utility of waste, rather than categorizing it into specific types. For example, rather than distinguishing whether waste is a banana or an apple, we focus on whether it can be composted. Similarly, the distinction between a shipping box and product packaging is less important than knowing whether the material is recyclable. This approach is illustrated in Figure 16, where the MRS-YOLO model’s capability to recognize different forms of kitchen waste is showcased-be it a whole apple, half an apple, an apple core, or a rotten apple. This capability is crucial for cities enforcing food waste classification policies as it enhances the efficiency and effectiveness of waste treatment processes and promotes higher rates of reuse.

**Figure 17:**
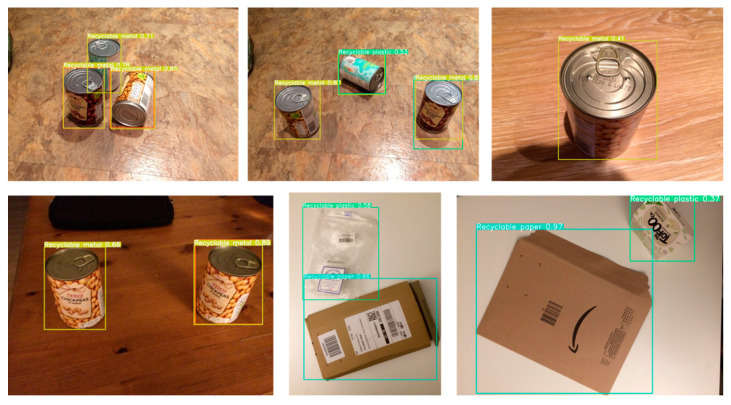
Detection effects at different angles.

For the detection effect of the same category under different angles and lighting effects, as shown in Figure 17, for the same type of items, whether placed vertically or lying flat, facing different light source positions and light source intensities, this model can still obtain good detection results.

**Figure 18:**
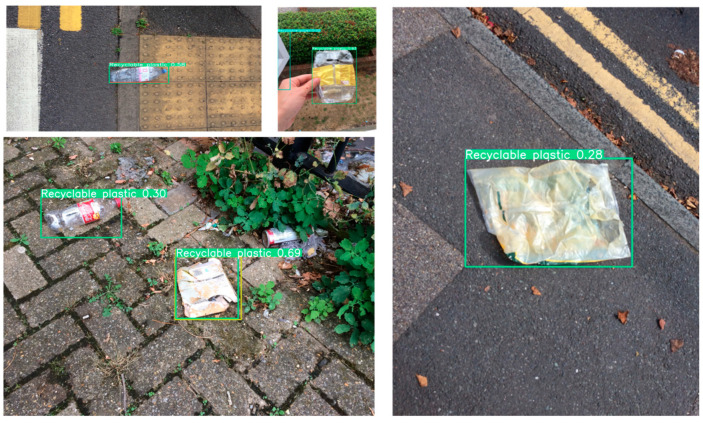
Detection results against a city street background.

**Figure 19:**
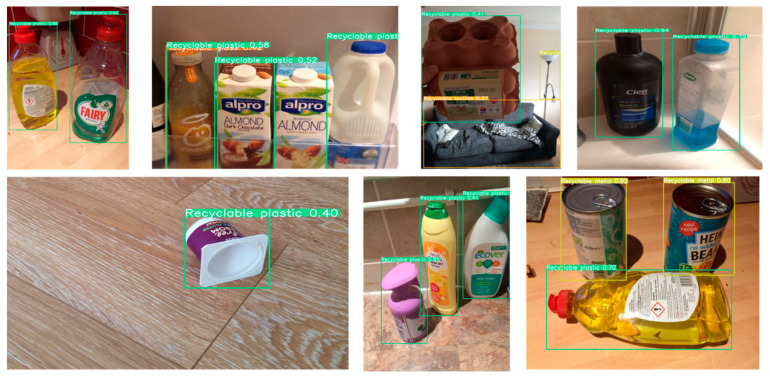
Detection effects in home background.

**Figure 20:**
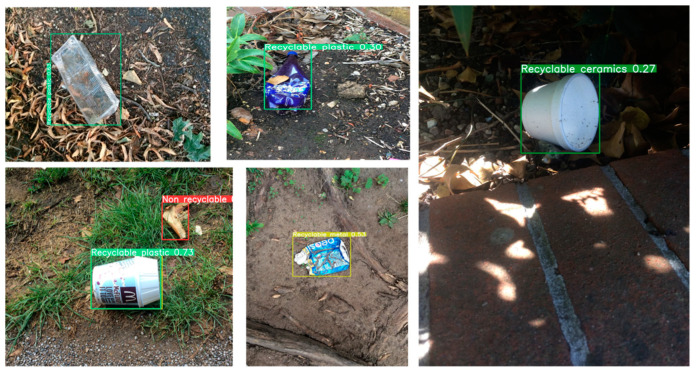
Detection effects under natural environment background.

In order to verify the detection ability of this model in different environments, in the background of urban street scenes (as shown in Figure 18), different types of plastic waste of different shapes can be accurately detected. In the home background (as shown in Figure 19), facing different light and different appearances of the same category of goods, this model can accurately detect. It is worth noting that in the third picture on the left of the first column of the figure, even if it is not in the focus area of the photo, MRS-YOLO improves the detection ability of small targets thanks to SlideLoss_IOU, so that it still detects the table lamp in the image as recyclable metal waste. In the background of the natural environment (as shown in Figure 20), even if the garbage has a larger deformation than the usual garbage shape, such as the second picture in the first column and the second picture in the second column, even with such deformation, this model can still detect that its category is recyclable plastic and recyclable metal, respectively. Even in the last large picture, facing such a dim environment, it can still detect that it is a ceramic flower pot.

In summary, the MRS-YOLO model has demonstrated strong performance and stability in multi-environment garbage detection tasks, and has demonstrated excellent target detection capabilities in complex scenarios. This highlights its potential for significant impact in automatic garbage sorting and environmental monitoring, making it a valuable tool for urban management and ecological protection.

## 5. Discussion

The MRS-YOLO model holds significant potential for deployment in real-world waste management systems due to its high precision and robust performance. However, implementing this model in practical applications involves addressing several challenges and considerations. The computational demands of the MRS-YOLO model necessitate the use of high-performance hardware, including GPUs with substantial memory capacity and high-speed processing capabilities to ensure real-time detection and classification. Potential solutions include the deployment of cloud-based GPU servers or edge devices with embedded AI capabilities to handle on-site processing. Ensuring real-time processing is crucial for effective waste management, especially in dynamic environments such as recycling centers and public waste collection systems. The model’s optimized architecture, including the integration of CP, SlideLoss_IOU, and RepViT, enhances its processing speed. However, further optimization techniques, such as model pruning and quantization, can be explored to improve inference speed without compromising accuracy.

Integrating the MRS-YOLO model with existing waste management systems involves addressing software and hardware interoperability. The model needs to communicate seamlessly with various sensors, data collection systems, and operational control units. Developing APIs and middleware that facilitate smooth data exchange and real-time updates can address this challenge. Additionally, establishing standardized protocols for data handling and system communication will ensure seamless integration.

To illustrate the practical utility of the MRS-YOLO model, consider the following hypothetical case studies that simulate its application in real-world scenarios.

In urban recycling centers, the MRS-YOLO model can be deployed to automate the sorting process of recyclable materials. Equipped with high-definition cameras, the system captures images of waste items on conveyor belts. The model detects and classifies each item in real-time, directing them to appropriate sorting bins. This automation significantly reduces manual labor, enhances sorting accuracy, and increases overall efficiency. By continuously monitoring the system’s performance, operators can identify bottlenecks and optimize operational workflows.

In a city-wide waste management initiative, the MRS-YOLO model can be integrated into smart waste bins placed in public areas. These bins, equipped with cameras and IoT sensors, utilize the model to identify and classify deposited waste items. The classification data are sent to a central management system, which provides real-time insights into waste composition and collection patterns. This information helps in optimizing collection routes, reducing operational costs, and promoting recycling efforts. Challenges such as varying lighting conditions and environmental factors can be mitigated through the model’s robust detection capabilities.

In industrial settings, the MRS-YOLO model can be used to manage waste generated from manufacturing processes. By integrating the model with robotic arms and conveyor systems, waste items are automatically identified and sorted based on material type. This automation minimizes human intervention, reduces sorting errors, and improves the efficiency of waste-handling processes. Regular performance evaluations and system updates ensure that the model adapts to changing waste types and quantities, maintaining high accuracy and reliability. These case studies demonstrate the practical advantages of the MRS-YOLO model in diverse waste management applications. The model’s ability to accurately detect and classify waste in real-time, coupled with its adaptability to different environments, underscores its potential to revolutionize waste management systems and contribute to environmental sustainability.

## 6. Conclusion and Future Research

This study introduces the MRS-YOLO model for high-precision waste detection and classification, showcasing its significant advancements over existing models. The key contributions include the novel feature extraction method based on C2f, the SlideLoss_IOU technique for improved small target detection, and the integration of the RepViT network architecture. These innovations collectively enhance the model’s accuracy, speed, and robustness. The comprehensive experimental validation, including ablation studies, highlights the effectiveness of each component and the synergistic improvements in performance. The practical applications and case studies further underscore the model’s potential to transform waste management systems.

While the MRS-YOLO model demonstrates substantial improvements, there are several areas for future research. Further optimization of the model, including techniques like model pruning and quantization, can be explored to enhance real-time processing capabilities without sacrificing accuracy. Expanding the application of the MRS-YOLO model to other waste management scenarios, such as marine debris detection and hazardous waste identification, can broaden its impact. Investigating solutions to overcome current limitations, such as performance under extreme environmental conditions or the detection of highly occluded objects, can further improve the model’s robustness. Developing seamless integration strategies with IoT-based waste management systems to facilitate real-time data exchange and operational control is also crucial. Finally, implementing long-term monitoring and evaluation frameworks to ensure the system’s stability, reliability, and adaptability to changing waste management needs will be vital. These future research directions aim to build upon the foundational work presented in this study, driving continued innovation and improvement in intelligent waste management technologies.

## Figures and Tables

**Table 1:** Comparison with YOLO series model accuracy.

Model	Backbone	mAP_0.5_/%	mAP_0.5:0.95_/%	Image Size	Parameters	FLOPs	Speed
YOLOv5n	Darknet-53 (C3)	70.9	55.21	640 × 640	2.64 M	7.1 G	8.5 ms-f^−1^
RT-DETR	Rtdetr-r18	69.6	59.1	640 × 640	19.00 M	57 G	2.8 ms-f^−1^
YOLOv8n	Darknet-53 (C2f)	68.6	58.5	640 × 640	3.01 M	8.7 G	5.8 ms-f^−1^
YOLOv3	Darknet-53	53.4	42.1	640 × 640	8.00 M	13 G	3.8 ms-f^−1^
YOLOv6n	EfficientRep	70.1	57.4	640 × 640	4.23 M	11.8 G	2.4 ms-f^−1^
YOLOv5s	CSP Darknet53	67.5	55.7	640 × 640	5.10 M	10.8 G	3.4 ms-f^−1^
Fast-RCNN [36]	ResNet101	64.2	61.7	640 × 640	4.8 M	19.1 G	53.2 ms-f^−1^
MASK-RCNN [36]	ResNet50	58.1	47.2	640 × 640	4.4 M	13.2 G	65.3 ms-f^−1^
Efficientdet [36]	EfficientNet-B2	63.8	60.4	640 × 640	9.3 M	10.1 G	17.5 ms-f^−1^
Ours	Darknet-53 (CP)	74.5	65.5	640 × 640	4.33 M	7.4 G	2.7 ms-f^−1^

**Table 2:** Ablation experiments.

+CP	+SlideLoss_IOU	+RepViT	mAP_0.5_/%	mAP_0.5:0.95_/%	Calculations/GFLOPs	Number of Participants/M	FPS (frame/s)
			70.9	61.6	8.1 G	3.0	5.8 ms-f^−1^
√			71.7	60.3	7.0 G	3.7	1.1 ms-f^−1^
√	√		73.9	62.5	7.0 G	3.7	5.3 ms-f^−1^
√	√	√	74.5	65.5	7.4 G	4.3	2.7 ms-f^−1^

**Table 3:** Comparison of the effect of feature extraction modules.

Model	Backbone	mAP_0.5_/%	mAP_0.5:0.95_/%	Image Size	Parameters	FLOPs	Speed
YOLOv5 (C3)	C3	62.8	57.6	640 × 640	2.50 M	7.2 G	1.6 ms-f^−1^
YOLOv5 (C2f)	C2f	69.6	58.4	640 × 640	2.50 M	7.1 G	1.1 ms-f^−1^
YOLOv5 (CP)	CP	72.8	56.9	640 × 640	3.78 M	6.9 G	1.2 ms-f^−1^

## Data Availability

The data supporting the reported results are publicly available and can be found at the following link: https://github.com/YizheDev/MRS−YOLO.git (accessed on 29 June 2024).

## Appendix A. Dataset Usage and Model Training Steps

(a) Visit the following link to download the required dataset: Dataset.
(b) After downloading, use the following command to extract the dataset: git clone https://oauth2:znaQ_T5zcUDQXkF8SVMn@www.modelscope.cn/datasets/YizheDev/trash_detection.git (accessed on 29 June 2024).
(c) The extracted dataset directory structure is as follows:

trash_detection/

├── train/

│ ├── images/

│ └── labels/

├── val/

│ ├── images/

│ └── labels/

└── test/

├── images/

└── labels/.

(d) Before training the model, please modify the configuration files data.yaml and MRS-YOLO.yaml according to your local environment, including the dataset path and training parameters.
(e) Use the following command to start training the model: yolo detect train data = /home/moxing/ultralytics/cfg/datasets/new_data/data.yaml model = /home/moxing/ultralytics/cfg/models/lunwen/RepVi+MCA+loss.yaml epochs = 300 imgsz = 640 resume = True workers = 16 device = 0.
(f) The main training parameters are described as follows:
  - data: Path to the dataset configuration file;
  - model: Path to the model configuration file;
  - epochs: Number of training epochs;
  - imgsz: Input image size;
  - resume: Whether to resume training from the last checkpoint;
  - workers: Number of data loading workers;
  - device: Device ID for training.
(g) After training, use the following command to validate the model performance: yolo detect val data = datasets/data.yaml model = runs/detect/train11/weights/best.pt.
(h) The final training results will be saved in the runs folder.
